# A lytic phage to control multidrug-resistant avian pathogenic *Escherichia coli* (APEC) infection

**DOI:** 10.3389/fcimb.2023.1253815

**Published:** 2023-09-07

**Authors:** Lan Yao, Yinli Bao, Jiangang Hu, Beibei Zhang, Zhiyang Wang, Xinyu Wang, Weiqi Guo, Di Wang, Jingjing Qi, Mingxing Tian, Yanqing Bao, Haihua Li, Shaohui Wang

**Affiliations:** ^1^ Shanghai Veterinary Research Institute, Chinese Academy of Agricultural Sciences, Shanghai, China; ^2^ Tianjin Key Laboratory of Agricultural Animal Breeding and Healthy Husbandry, College of Animal Science and Veterinary Medicine, Tianjin Agricultural University, Tianjin, China; ^3^ Engineering Research Center for the Prevention and Control of Animal Original Zoonosis of Fujian Province University, College of Life Science, Longyan University, Fujian, China

**Keywords:** avian pathogenic Escherichia coli, multidrug-resistant, bacteriophage, characteristics, biofilm

## Abstract

The inappropriate use of antibiotics has led to the emergence of multidrug-resistant strains. Bacteriophages (phages) have gained renewed attention as promising alternatives or supplements to antibiotics. In this study, a lytic avian pathogenic *Escherichia coli* (APEC) phage designated as PEC9 was isolated and purified from chicken farm feces samples. The morphology, genomic information, optimal multiplicity of infection (MOI), one-step growth curve, thermal stability, pH stability, *in vitro* antibacterial ability and biofilm formation inhibition ability of the phage were determined. Subsequently, the therapeutic effects of the phages were investigated in the mice model. The results showed that PEC9 was a member of the siphovirus-like by electron microscopy observation. Biological characterization revealed that it could lyse two serotypes of *E. coli*, including O1 (9/20) and O2 (6/20). The optimal multiplicity of infection (MOI) of phage PEC9 was 0.1. Phage PEC9 had a latent period of 20 min and a burst period of 40 min, with an average burst size of 68 plaque-forming units (PFUs)/cell. It maintained good lytic activity at pH 3-11 and 4-50°C and could efficiently inhibit the bacterial planktonic cell growth and biofilm formation, and reduce bacterial counts within the biofilm, when the MOI was 0.01, 0.1, and 1, respectively. Whole-genome sequencing showed that PEC9 was a dsDNA virus with a genome of 44379 bp and GC content of 54.39%. The genome contains 56 putative ORFs and no toxin, virulence, or resistance-related genes were detected. Phylogenetic tree analysis showed that PEC9 is closely related to *E. coli* phages vB_EcoS_Zar3M, vB_EcoS_PTXU06, SECphi18, ZCEC10, and ZCEC11, but most of these phages exhibit different gene arrangement. The phage PEC9 could successfully protect mice against APEC infection, including improved survival rate, reduced bacterial loads, and organ lesions. To conclude, our results suggest that phage PEC9 may be a promising candidate that can be used as an alternative to antibiotics in the control of APEC infection.

## Introduction

Avian pathogenic *Escherichia coli* (APEC) can cause colibacillosis in poultry, resulting in systemic infection and characteristic fibrous lesions such as perihepatitis, pericarditis, and airsacculitis (Guabiraba and Schouler, 2015), which have brought detrimental economic losses in the poultry industry worldwide. It has been reported that APEC is a reservoir of drug-resistance genes and virulence genes of human extraintestinal pathogenic *E. coli* (ExPEC), posing a major threat to human health and public health (Tivendale et al., 2010; Mellata, 2013). Among the various serotypes of APEC O1, O2, and O78 are the most prevalent serogroups (Wang et al., 2014; Kathayat et al., 2021). Historically, antibiotics have been the primary approach for preventing and control of APEC infection. However, the long-term unreasonable use of antibiotics has led to the emergence of a large number of multi-drug-resistant bacteria, including APEC (Frieri et al., 2017; Qiao et al., 2018; Roth et al., 2019). APEC can form biofilms on biotic or abiotic surfaces to protect bacterial cells against harm. It was indicated that biofilm enhance bacterial resistance to antibiotics (Venkatesan et al., 2015). Consequently, there is an urgent need to develop alternative approaches to prevent and control bacterial infections.

Phages, which are the most abundant living entities on earth, are viruses that exclusively infect bacteria (Clokie et al., 2011). They have been used to treat bacterial infections since their discovery more than a century ago (Bach-Rojecky and Lacković, 2021). In comparison with antibiotics, phages show many advantages in dealing with bacterial infections such as host specificity, fast proliferation, high safety, and low cost. Particularly, the increasing emergence of antibiotic-resistant bacteria has led to a resurgence of interest in phage therapy (Kutateladze and Adamia, 2010; Sharma et al., 2017). Phage therapy has been widely used to prevent and control pathogenic bacteria in various fields such as medicine, animal husbandry and veterinary medicine, and food hygiene (Sarhan and Azzazy, 2015; Caflisch et al., 2019).

In this study, a lytic APEC phage PEC9 was isolated, and its biological characteristics and genome sequence were analyzed. The potential use of PEC9 phage in the biocontrol of colibacillosis was investigated.

## Materials and methods

### Bacterial strains and growth conditions

The bacterial strains, containing 10 *Salmonella* strains, 58 *E. coli* strains, and 10 *Staphylococcus aureus* strains, were isolated from chicken farms in eastern China through selective medium culture and PCR confirmation in our previous studies (Wang et al., 2019; Tao et al., 2021; Afayibo et al., 2022). These bacteria were used for phage isolation and lytic spectrum determination. All bacteria were grown in Luria-Bertani (LB) broth at 37°C.

### Isolation and purification of bacteriophage

A total of 50 chicken fecal samples were collected from chicken farms in Shanghai, China. Isolation and purification of phages were performed as previously described (Manohar et al., 2018; Kazibwe et al., 2020) with some modifications. Briefly, particulate matter in the samples was removed by centrifugation at 1000 × *g*. The enriched culture was centrifuged at 7000 × *g* for 10 min at 4°C, and the supernatant was filtered using a 0.22-μm filter membrane (Millipore, USA) to remove bacteria. The filtrate was mixed with the host APEC AH50 (OD_600 = _0.6 ~0.8) and incubated at 37°C 120 rpm to enrich phages. The enriched culture was then filtered. Equal volumes of the filtrate and the host AH50 were mixed with melted semisolid medium (0.7% agarose), spread onto LB plates according to the double-agar overlay method, and incubated overnight at 37°C. Purified phages were obtained by performing the plaque assay six times and stored at 4°C for further studies.

### Phage morphology

Phage morphology was analyzed through transmission electron microscopy (TEM). The filtered high-titer phage particles were dropped on a copper grid and negatively stained with 2% phosphotungstic acid. Excess liquid was blotted off and then the grids were observed under a FEI T12 transmission electron microscope (FEI, Ltd, Hillsboro, OR, USA).

### Determination of phage lytic spectrum

The lytic spectrum of phage PEC9 was determined by the spot test and double-agar overlay method according to the previous study (Sasikala and Srinivasan, 2016).

### MOI assay of phage

The optimal MOI of phage PEC9 was determined as previously described (Lu et al., 2003) with some modifications. The host strain AH50 was mixed with diluted phage (1 × 10^5^ - 1 × 10^10^ PFUs/mL) at the MOI as 0.001, 0.01, 0.1, 1, 10, and 100. Mixtures were cultured at 37°C 120 rpm for 4 h and then filtered through a 0.22-µm filter. The phage titers were determined using the double-agar overlay method. The experiments were performed independently three times.

### Phage adsorption rate assay

The adsorption ability of phage PEC9 was determined as previously described (Al-Zubidi et al., 2019)with some modifications. Briefly, phage PEC9 was mixed with host strain AH50 at an MOI of 0.1 and incubated at 37°C 120 rpm. Samples were taken every 2 min for 12 min and centrifuged to remove the absorbed phages. Finally, the titers of unabsorbed phages in the supernatant were determined after serial dilution. The percentages of phage adsorption at different time points were calculated as follows: [(initial phage titer-free phage titer in supernatant)/initial phage titer] ×100.

### One-step growth curve assay

A one-step growth curve assay was performed as previously described with some modifications (Li et al., 2021). Phage PEC9 was mixed with AH50 at an MOI of 0.1 and incubated at 37°C 120 rpm for 10 min to maximize phage adsorption. Subsequently, the mixture was centrifuged to remove unabsorbed phage. The sediment was washed three times in PBS and resuspended with 10 mL of pre-warmed LB broth, then incubated for 130 min. During this period, the samples were taken every 10 min. The phage titers were determined by the double-agar overlay method. The experiments were performed independently three times.

### Thermal and pH stability

For thermal stability testing, the phage suspension was incubated at 4, 37, 50, 60, 70, and 80°C for 1 hour, respectively. The samples were taken after 20, 40, and 60 min and the phage titers were determined. In addition, the phage suspension was stored at room temperature (25°C) for 4 weeks and the phage titers were determined every week.

For pH stability testing, the phage suspension was added to PBS buffer at pH ranging from 2 to 13 (adjusted with HCl or NaOH for acidic or alkaline, respectively) and incubated at 37°C for 1 h. The viable phages in each pH environment were detected by the double-agar overlay method.

### Inhibition of planktonic bacterial cells by phages *in vitro*

The bacterial challenge test of the phage was performed as previously described with some modifications (Tao et al., 2021). The host strain AH50 grew to an OD_600nm_ of 0.2 and was infected with phage PEC9 at the MOI as 0.01, 0.1, and 1 respectively, followed by incubation at 37°C 120 rpm for 12 h. AH50 cultured without phage PEC9 was used as the control group. Bacterial growth was determined by monitoring the OD_600nm_ at 2 h intervals. Meanwhile, the number of viable bacterial cells were determined by LB plate counting. The experiments were performed independently three times.

### Inhibition of bacterial biofilm by phages

The inhibition effect of phage PEC9 on biofilm formation by APEC AH50 was tested by referring to previous studies (Fong et al., 2017; Lajhar et al., 2018; Chaudhary et al., 2022). The host AH50 and phage PEC9 were inoculated into 96 well plates at the MOI of 0.01, 0.1, and 1 respectively, and cultured without shaking at 37°C for 24 h. Planktonic bacterial cells were removed by rinsing with PBS buffer. After air-drying, the biofilms in the wells were stained with crystal violet (0.2%, W/V) for 20 min. The biofilms were solubilized with 95% absolute ethanol and the absorbance was measured at 595 nm. The experiments were repeated in duplicate.

The effect of phage PEC9 on the number of bacteria within the biofilm was tested as described previously (Dickey and Perrot, 2019; Duc et al., 2020) with some modifications. After 24 h incubation, 96 well plates were washed twice with PBS to remove the planktonic bacterial cells. The biofilms in the wells were disrupted with pipet tips and suspended in pre-cooled PBS. The bacteria were enumerated by plating. The experiments were repeated in duplicate.

### Phage genome sequencing and analysis

Phage DNA was extracted using the Phage Genome DNA Quick Extraction Kit (Zhuangmeng International Biology Gene Technology Co., Ltd). DNA concentration was determined using a spectrophotometer (Nanodrop Technologies, USA). The PEC9 genomic DNA was sequenced using an Illumina NovaSeq PE150 sequencer and reads were assembled into a whole genome using SOAPdenovov 2.04 software and GapCloserv1.12. High-quality paired-end reads were assembled using A5-MiSeq v20160825 (https://arxiv.org/abs/1401.5130) and SPAdes v3.12.0 (http://cab.spbu.ru/files/release3.12.0/manual.html), and the genome sequence was proofread using software MUMmer v3.1 (http://mummer.sourceforge.net/) (Kurtz et al., 2004) and Pilon v1.18 (https://github.com/broadinstitute/pilon) (Walker et al., 2014). Potential open reading frames (ORFs) were predicted using GeneMarkS v4.32 (Besemer et al., 2001). Genome annotation was analyzed using diamond vO.8.36 (http://github.com/bbuchfink/diamond), HHpre (https://toolkit.tuebingen.mpg.de/#/tools/hhpred), BLAST and Conserved Domain Identify of NCBI. The Virulence Factor Database (http://www.mgc.ac.cn/VFs/main.htm) and Comprehensive Antibiotic Resistance Database (https://card.mcmaster.ca/) were queried to retrieve the toxic genes, virulence genes, and antibiotic resistance genes in the phage genome. tRNAscan-SE search program (https://lowelab.ucsc.edu/tRNAscan-SE/) was used to identify putative tRNAs (Lowe and Chan, 2016). A circular representation of the genome of phage PEC9 was generated using BRIG software. Comparisons and phylogenetic analysis of the genome of phage PEC9 with other phages were conducted with the NCBI BLASTN algorithm (http://blast.ncbi.nlm.nih.gov).

### Efficacy of phage therapy for APEC infection in mouse model

The care and maintenance of all animals were performed following the guidelines of the Institutional Animal Care and Use Committee of Shanghai Veterinary Research Institute, Chinese Academy of Agricultural Sciences (CAAS). The Ethics Committee of CAAS approved the use of mice for this study. The permit was documented under the number SHVRI-SV-20220812-G01.

To study the therapeutic effect of phage on APEC infection in mice. Twenty-four 6-week-old specific-pathogen-free (SPF) BALB/c mice were divided randomly into four groups (infection group, treatment group 1, treatment group 2, control group), with six mice in each group for the experiment (n=6). The mice were housed in SPF mice isolator and had free access to food and water during the study period. All mice in four groups were infected with AH50 of 0.2 mL by intraperitoneal administration. The treatment group 1 was administered with 0.2 mL (1.0 × 10^8^ PFUs) (MOI=1) of phage PEC9 intraperitoneally at the 6 h post-infection. Treatment group 2 was injected intraperitoneally with 0.2 mL (1.0 × 10^8^ PFUs) of phage PEC9 at 12 h post-infection. The control group was challenged with PBS buffer. The mice were monitored daily for 14 days to calculate the survival rate.

To evaluate the therapeutic efficacy of phage PEC9 *in vivo*, twenty 6-week-old SPF BALB/c mice were divided randomly into four groups (infection group, treatment group 1, treatment group 2, control group) with five mice in each group for the experiment (n=5). All mice in four groups were infected with AH50 of 0.2 mL by intraperitoneal administration. The treatment group 1 was administered 0.2 mL (1.0 × 10^7^ PFUs) of phage PEC9 intraperitoneally at the 6 h post-infection. Treatment group 2 was injected intraperitoneally with 0.2 mL (1.0 × 10^7^ PFUs) of phage PEC9 at 12 h post-infection. The control group was challenged with PBS. The live mice were euthanized at 24 h post-infection. Spleens were taken aseptically, weighed, homogenized in sterile PBS, and inoculated on LB agar plates to determine the bacteria counts. The livers and spleens were removed under sterile conditions and were fixed in 4% paraformaldehyde fix solution for pathological studies. The specimen was embedded and sliced, and then stained with hematoxylin and eosin (H&E).

### Statistical analyses

Statistical analyses were conducted using the GraphPad Prism software (version 6.0) package. Multivariate comparisons were analyzed by using one-way or two-way analysis of variance (ANOVA). A value of *P* < 0.05 was considered statistically significant.

## Results

### Isolation, purification, and morphology of phage

**Figure 1:**
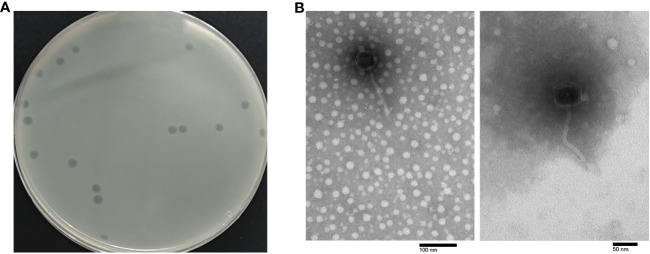
Morphology of phage PEC9. **(A)** Plaque morphology of phage PEC9. **(B)** Transmission electron micrographs of phage PEC9. Scale bar, 50 nm or 100 nm, respectively.

Phage PEC9 was isolated and purified from chicken feces using APEC strain AH50 of serotype O1 as the host. It formed transparent round plaques on the double-layer agar plate with a diameter of 3.5-4 mm and clear boundaries (**Figure 1A**).

TEM showed that phage PEC9 was composed of an icosahedral symmetry head with a diameter of 54.2 ± 0.8 nm and a non-contractile long tail of 128.4 ± 1.6 nm (**Figure 1B**). The morphology of the phage indicated that it belonged to the class *Caudoviricetes* and T-5 like according to the guidelines of the International Committee on Taxonomy of Viruses (Zhu et al., 2022).

### Lytic spectrum determination of phage PEC9

The lytic spectrum of phage PEC9 was determined by the spot test and double-agar overlay method. PEC9 showed lytic activity against two serotypes of *E. coli*, including O1 (9/20) and O2 (6/20) (**Table 1**). The phage had no infective activity against *S.* Pullorum, *S.* Gallinarum, *S.* Enteritidis, and *Staphylococcus aureus.*

**Table 1:** The lytic spectrum of phage PEC9.

Bacteria	Serotype	No. of total strain	No. of lysed by phage PEC9
*Escherichia coli*	O1	20	9
*Escherichia coli*	O2	20	6
*Escherichia coli*	O18	2	0
*Escherichia coli*	O78	8	0
*Escherichia coli*	O8	3	0
*Escherichia coli*	O9	5	0
*Salmonella*	*S.* Pullorum, *S.* Gallinarum, *S.* Enteritidis	10	0
*Staphylococcus aureus*		10	0

### Optimal MOI of phage PEC9

The host bacteria APEC AH50 was infected with phage PEC9 at various ratios, and the phage titer was tested to determine the optimal MOI. When the MOI was 0.1, PEC9 obtained the highest titer, indicating that the optimal MOI was 0.1 (**Table 2**).

**Table 2:** Optimal MOI of phage PEC9.

MOI	Bacterial concentration (CFU/mL)	Phage titer (PFU/mL)	Phage titer after incubation (PFU/mL)
0.001	1×10^8^	1×10^5^	4.183×10^8^
0.01	1×10^8^	1×10^6^	1.216×10^9^
0.1	1×10^8^	1×10^7^	3.500×10^9^
1	1×10^8^	1×10^8^	1.107×10^9^
10	1×10^8^	1×10^9^	3.583×10^8^
100	1×10^8^	1×10^10^	5.517×10^8^

### One-step growth curve

**Figure 2:**
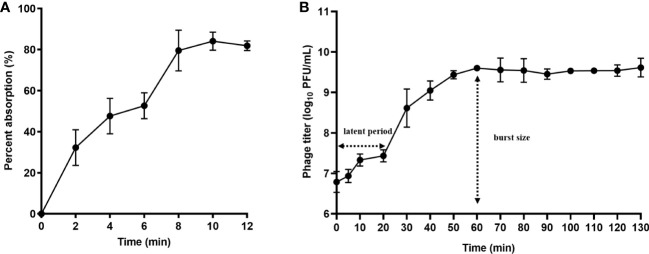
Adsorption rate and one-step growth curve of phage PEC9. **(A)** Adsorption rate. Adsorption of phage PEC9 to APEC AH50 was expressed as a percentage of the total phages added. **(B)** One-step growth curve of phage PEC9 on APEC AH50.

The results showed that phage PEC9 had an adsorption rate of 33.8% within 2 min, 52.7% within 6 min, 79.6% within 8 min, 84.1% within 10 min, and 81.9% within 12 min, indicating that adsorption reached saturation after about 10 min (**Figure 2A**). This result indicated the high and rapid adsorption rate of phage PEC9.

According to the one-step growth curve **(** **Figure 2B** **)**, phage PEC9 had a latent period of about 20 min and a burst period of 40 min, with an average burst size of 68 PFUs/cell. The phage reached the stationary phase after 60 min.

### Thermal and pH stability of phage PEC9

**Figure 3:**
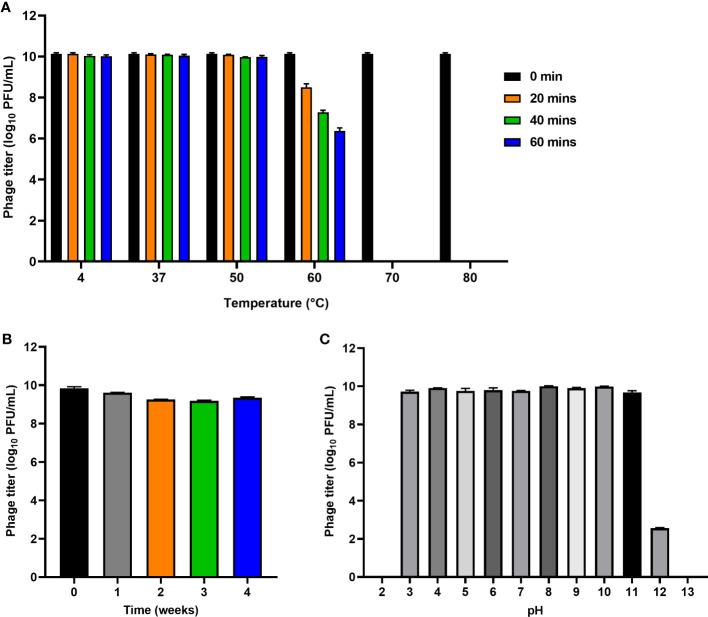
Stability of phage PEC9 under different temperature and pH. **(A)** Thermal stability test. **(B)** Room temperature storage test. **(C)** pH stability test.

The stability of phage PEC9 was tested under varied conditions. Thermal stability tests indicated that PEC9 could survive stably in a wide temperature range of 4-50°C (**Figure 3A**). However, the phage titer was gradually decreased when the incubation temperature was above 50°C and the phage was completely inactivated when the temperature was raised to 70°C for 20 min. In addition, Phage PEC9 could maintain stable activity for 4 weeks at room temperature (**Figure 3B**), indicating its ability to tolerate normal temperature environments and its therapeutic potential in practical application.

Phage PEC9 was found to be stable in the pH range of 3~11 (**Figure 3C**), suggesting its ability to tolerate extreme environments. The titer was dramatically decreased at pH 12 and no phages survived at pH 2 or pH 13.

### Inhibition of APEC planktonic cells by phage PEC9 *in vitro*

**Figure 4:**
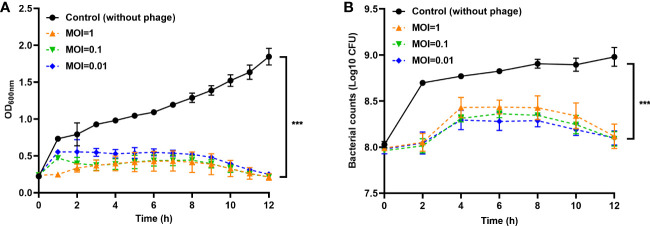
Inhibition of the host planktonic cells by phage PEC9 *in vitro*. The host AH50 was incubated with or without phage PEC9. The growth **(A)** and bacterial counts **(B)** of AH50 were inhibited by phage PEC9. Statistical significance was assessed using one-way ANOVA (***P < 0.001).

To evaluate the antibacterial effect of phage PEC9 *in vitro*, the host AH50 was infected with PEC9 at 3 different MOIs. As shown in **Figure 4A**, phage PEC9 could constantly inhibit the growth of the host at MOI of 0.01, 0.1, and 1 in 12 h. Meanwhile, the number of viable cells of three groups (MOI=0.01, 0.1, and 1) treated with phages was decreased to 0.12 log10 CFU/mL, 0.11 log10 CFU/mL, and 0.14 log10 CFU/mL, respectively, at 12 h compared with the control group (**Figure 4B**).

### Control of biofilm formation by phage PEC9

**Figure 5:**
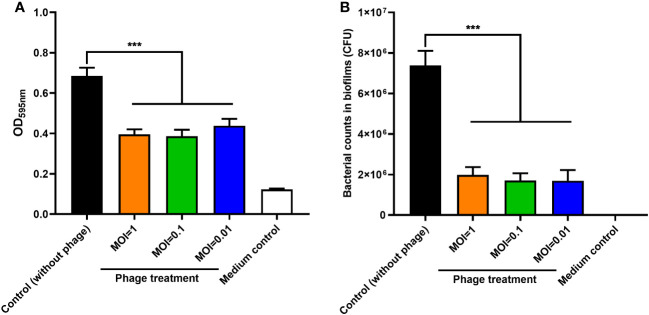
The effect of phage PEC9 on biofilm formation by host AH50 was tested. **(A)** The bacterial biofilm formation was significantly inhibited when treated with different MOIs of phage PEC9. **(B)** The bacterial counts within the biofilm were significantly inhibited by different MOIs of phage PEC9. Statistical significance was assessed using one-way ANOVA (*** *P* < 0.001).

The effect of phage PEC9 on inhibiting biofilm formation was determined by crystal violet staining in 96-well plates. Compared with the control group, the biofilm formation of APEC AH50 was significantly inhibited by phage PEC9 when MOI=0.01, 0.1, and 1 (**Figure 5A**) (*P* < 0.001). And the bacterial viable counts within the biofilm were significantly reduced by phage PEC9 at different MOI (**Figure 5B**) (*P* < 0.001).

### Genome analysis of phage PEC9

**Figure 6:**
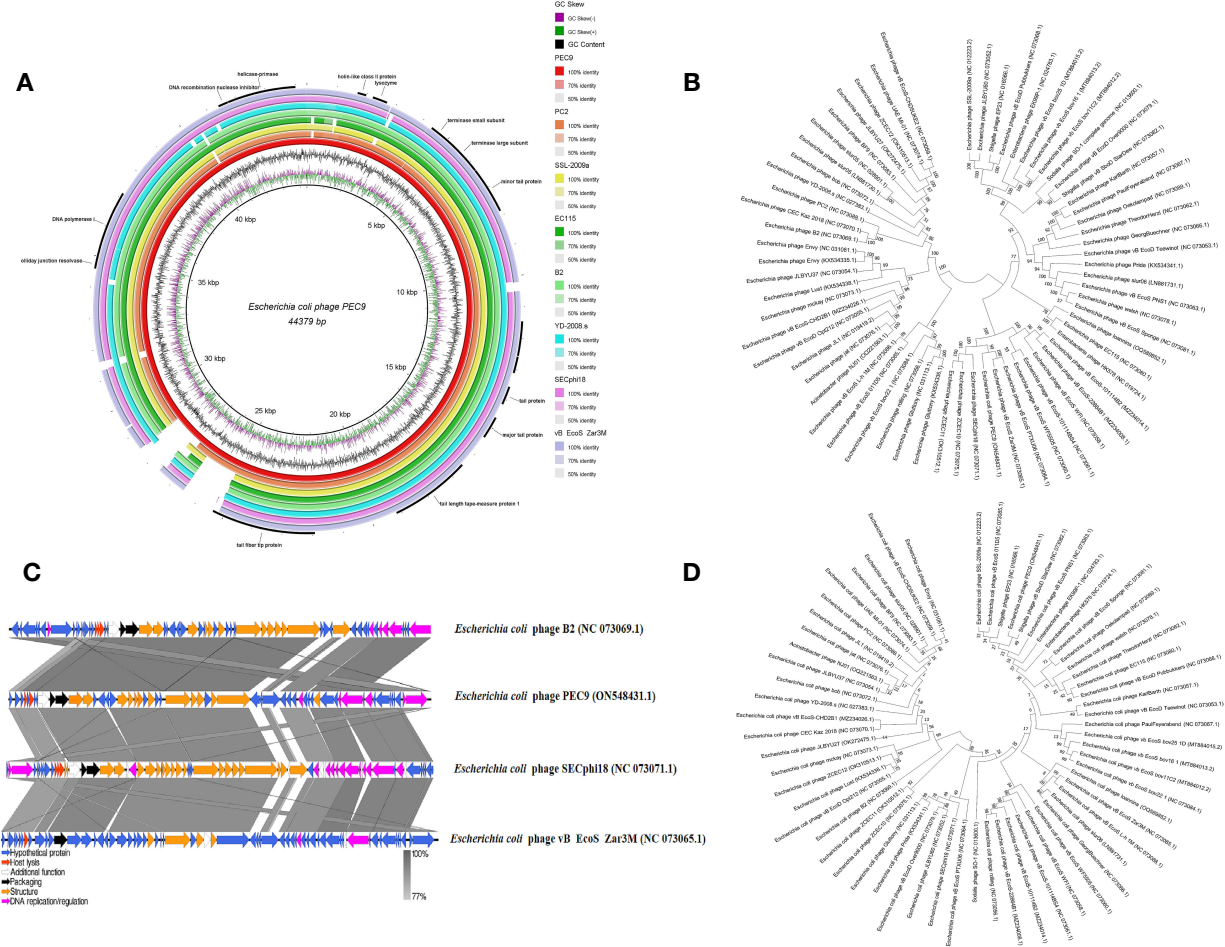
Phage PEC9 whole-genome analysis. **(A)** Circular genome map of phage PEC9. The genomic organization of phage PEC9 was compared to *E. coli* phages PC2, SSL-2009a, EC115, B2, YD-2008.s, SECphi18 and vB EcoS Zar3M. The coincident regions of other *E. coli* phages were displayed, and the blank was the non-coincident region. The outermost circle represents some important functional proteins of phage PEC9. **(B)** Neighbor-joining phylogenetic tree constructed based on the whole genome sequences of *E. coli* phages. **(C)** Linear comparison analysis between the whole genome of *E. coli* phages PEC9 and B2, SECphi18, and vB EcoS Zar3M. The shade of color in the middle shaded part indicates the degree of homology. Arrows indicate open reading frames transcribed in either the rightward or leftward direction. **(D)** A Neighbor-joining tree constructed based on the amino acid sequence of terminase large subunit.

Whole-genome sequencing revealed that the double-stranded DNA (dsDNA) of phage PEC9 consisted of 44379 bp with a GC content of 54.39% (**Figure 6A**). The genome annotation analysis indicated that PEC9 had 56 ORFs, of which 31 were located on the plus strand and the other 25 were located on the minus strand. Among the 56 ORFs, only 24 ORFs (42.9%) had annotated functions, and the other 32 ORFs (57.1%) were annotated as hypothetical proteins. The functional proteins were categorized into three groups, including DNA replication/metabolism-related proteins, structure/packaging proteins, and host lysis proteins. No tRNA genes were found in the phage genome, indicating that PEC9 is completely dependent on the host for protein synthesis. The integrase gene was not identified, suggesting that phage PEC9 should be a virulent phage. In addition, no toxin genes, virulence genes, and resistance genes were detected in the genome of phage using the Virulence Factor Database (http://www.mgc.ac.cn/VFs/main.htm) and Comprehensive Antibiotic Resistance Database (https://card.mcmaster.ca/), implying the safety of phage PEC9 in clinical application. The genome sequence of phage PEC9 has been deposited in the GenBank database under accession number ON548431.1.

### Comparative genomics and phylogenetic analysis of phage PEC9

Based on the result of BLAST analyses, the genome sequence of PEC9 displays significant similarity to many phages isolated from different regions around the world, suggesting that complex evolutionary relationships exist among these phages. The phylogenetic tree of these phages has two main branches (**Figure 6B**). Phylogenetic tree analysis showed that PEC9 is closely related to *E. coli* phages vB_EcoS_Zar3M (coverage 92%, identity 88%), vB_EcoS_PTXU06 (coverage 92%, identity 90%), SECphi18 (coverage 92%, identity 91%), ZCEC10 (coverage 94%, identity 91%) and ZCEC11 (coverage 94%, identity 91%). Comparisons of phage genomes were visualized using the Easyfig 2.2.5 tool. As shown in **Figure 6C**, multiple alignments of the PEC9 and relative phages showed that most of the regions are highly homologous at protein levels, but they exhibit different gene arrangements with each other. Compared with PEC9, some homologous genes of phages B2, vB_EcoS_Zar3M, and SECphi8 rearranged. According to the phylogenetic analysis of the large terminase subunit, PEC9 was clustered with the terminase of *Shigella* phage vB_SboD_StarDew, *Shigella* phage EP23, *E. coli* phage SSL-2009a, and *E. coli* phage vB_EcoS_011D5 (**Figure 6D**).

### The therapeutic effect of phage PEC9

**Figure 7:**
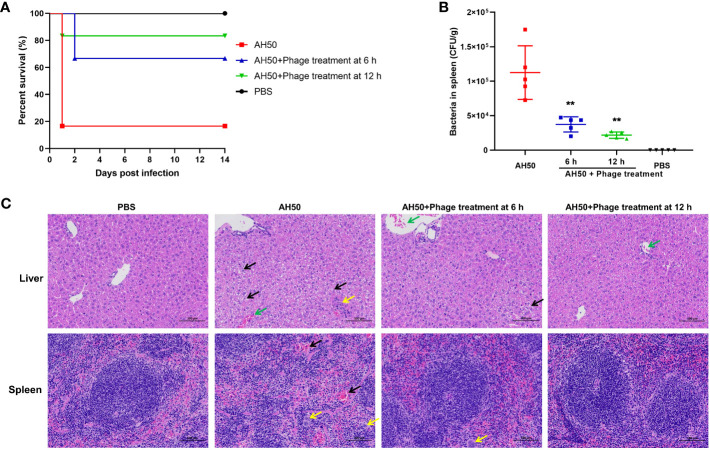
The therapeutic effect of phage PEC9 against APEC infection *in vivo*. **(A)** Survival rates of mice. The mice were administered intraperitoneally with phage PEC9 at 6 or 12 h post-infection. Mice injected with APEC AH50 or PBS only were used as the control groups. The survival rates of mice were monitored. **(B)** Bacteria concentrations in spleens of the phage-administered and control mice. The live mice were sacrificed at 24 h post-infection. (** *P* < 0.01). **(C)** Histopathological examination of mouse livers and spleens in 24 h after infection. From left to right, the pathological section results of livers and spleens of mice in the healthy group, the infection control group, the 6 h treatment group, and the 12 h treatment group. In the liver pathological micrographs, the black arrows indicate moderate watery degeneration of diffuse liver cells, swelling of liver cells, loose cytoplasm, and small vacuoles in the cytoplasm. The yellow arrow indicates necrotic foci in lobules, broken nuclei of hepatocytes, disintegration of cytoplasm, and minimal granulocyte infiltration. The green arrows indicate central vein congestion. In the spleen pathological micrographs, the black arrows indicate that the number of lymphocytes is reduced and the red pulp is moderately congested. The yellow arrows indicate that multinucleated giant cell increase.

The protective effect of phage PEC9 against APEC infection was tested in a mouse model. The mice were administered intraperitoneally with phage PEC9 at 6 or 12 h post APEC infection. The survival rate of the infection group was 16.7%, however, the survival rates of mice treated with phage PEC9 at 6 or 12 h post-infection were 66.7% or 83.3%, respectively (**Figure 7A**). This result showed that phage PEC9 could improve the survival rate of mice infected with APEC.

To evaluate the therapeutic efficacy of phage PEC9 *in vivo*, we counted the viable bacteria in the mice spleens. As shown in **Figure 7B**, the number of viable bacteria in the spleens of the phage treatment groups were significantly lower than that of the control group, and the effect of the 12 h treatment showed better effects. In addition, we observed the pathological changes in the liver and spleen tissues of phage-treated, control, and healthy mice (**Figure 7C**). There was diffuse moderate watery degeneration of hepatocytes in the liver tissues of infection control mice. Small necrotic foci were occasionally seen in the lobules, the nuclei of hepatocytes were fragmented and the cytoplasm was disintegrated. A small amount of granulocyte infiltration and central venous congestion were also observed. In contrast, in the livers of mice in the 6 h treatment group, occasional venous congestion and mild watery degeneration of hepatocytes were seen. The liver pathological changes of mice in the 12 h treatment group were slightly alleviated, close to those of healthy mice.

For spleen lesions, we observed that the capsule structure of spleen tissues in the control mice was not clear, with cell lysis, degeneration, and necrosis, as well as a decrease in lymphocytes, an increase in multinucleated giant cells, and moderate congestion of the red pulp. Compared with the AH50-control group, the capsule structure of spleen tissues in the treatment group was clear and normal, accompanied by an increase in multinucleated giant cells. These results indicated that phage PEC9 can effectively alleviate the liver and spleen lesions caused by APEC infection.

## Discussion

Avian colibacillosis caused by APEC results in huge economic losses to the poultry industry worldwide. APEC infections have traditionally been controlled by antibiotics. However, the unreasonable use of antibiotics leads to bacterial multi-drug resistance. Against this backdrop, phages as alternatives to antibiotics, have received extensive attention from scholars both domestically and internationally. The therapeutic effect of phage in mice infected with APEC has been evaluated (Wang et al., 2006).

In this study, a lytic APEC phage PEC9 was isolated and purified from chicken feces. It is necessary to conduct biological characteristics and whole-genome analysis before determining whether a phage can be a potential antimicrobial agent. Phage PEC9 can specifically lyse *E. coli* of O1 and O2 serotypes, with a cleaving rate of 33.3% and 13.3% respectively, showing stronger lysis ability compared to the *E. coli* phage isolated by Xu et al. (Xu et al., 2018). Electron microscopy observation and genome sequencing showed that PEC9 was a member of the class *Caudoviricetes*. *Caudoviricetes* (isometric head and non-contractile tail) is the most common phage group discovered to date. The optimal MOI of PEC9 was 0.1, suggesting that fewer phages can produce large numbers of phage progeny. The adsorption of phage particles to bacterial cells is the initial and key step in phage infection. About 84.1% of phage PEC9 adsorbed on the host within 10 min, indicating its high and rapid adsorption rate. The latent period, burst size, and stability of phage in different environments are key factors for the therapeutic applications of phages. Phages with larger burst sizes are beneficial for eliminating the targeted bacteria. PEC9 had a latent period of 20 min and an average burst size of 68 PFU/cell, which was different from phage JS09 with a latent period of 30 min and a burst size of 79 PFU/cell (Zhou et al., 2015). Phage PEC9 had a wide range of adaptability to temperatures ranging from 4 to 50°C and can retain stability at room temperature for four weeks. In addition, PEC9 was stable in the pH range of 3-11, showing its tolerance ability to extreme environments. These results suggested its suitability for practical applications.

APEC can form biofilms on biotic or abiotic surfaces, which contributes to antibiotic resistance and prolonged infections, making treatment more difficult. PEC9 efficiently inhibited the bacterial planktonic cell growth and biofilm formation, and reduce bacterial counts within the biofilm, when the MOI was 0.01, 0.1, and 1 respectively. The strong lytic ability of PEC9 suggested its potential to combat bacterial infections. The analysis of some biological properties of PEC9 provides a theoretical basis for its future applications. The bacterial counts within the mouse spleen and the survival rate of mice showed the protective effect of phage PEC9 on mice infected with APEC. It was also shown that different vaccination times affected the treatment efficacy. Pathological examination of mouse livers and spleens further confirmed the therapeutic effect of phage PEC9, reflecting the potential of this phage in the treatment of colibacillosis.

Whole-genome analysis is an effective way to further understand the characteristics of phage. Phages, as vectors for horizontal gene transfer, are probably a potential reservoir for antibiotic-resistant genes, virulence genes acquisition, and dissemination (Balcazar, 2014; Touchon et al., 2017). No toxin genes, virulence genes, resistance genes, and integrase genes were identified in the whole genome of phage PEC9 through some online databases, indicating its safety in clinical application as a bacterial control agent. Both whole-genome sequence alignment analysis and phylogenetic analysis based on the conserved terminate large subunit show that PEC9 is closely related to other phages. This suggested a complex evolutionary relationship between these phages. The results of the comparative genomic analyses extend our understanding of the evolution and relationship between PEC9 and its bacteriophage relatives.

In this study, we successfully isolated a novel APEC phage, PEC9, which could lyse two serotypes of *E. coli* and showed high tolerance to temperature and pH. Moreover, phage PEC9 had a strong lysis ability and significantly inhibited bacterial planktonic cell growth *in vitro*, biofilm formation, and bacterial counts within the biofilm of the host strain. Whole-genome analysis showed the safety of the phage in clinical applications. PEC9 exerted protective effects on mice infected with APEC. All these results suggest that phage PEC9 may be a candidate for the treatment of major prevalent serotypes of APEC infections.

## Data availability statement

The original contributions presented in the study are included in the article. Further inquiries can be directed to the corresponding authors.

## Ethics statement

The animal study was approved by The care and maintenance of all animals were performed following the guidelines of the Institutional Animal Care and Use Committee of Shanghai Veterinary Research Institute, Chinese Academy of Agricultural Sciences (CAAS). The Ethics Committee of CAAS approved the use of mice for this study. The study was conducted in accordance with the local legislation and institutional requirements.

## Author contributions

LY: Data curation, Validation, Visualization, Writing – original draft. YLB: Data curation, Validation, Writing – review & editing. JH: Data curation, Validation, Writing – review & editing, Funding acquisition, Project administration. BZ: Validation, Writing – review & editing. ZW: Validation, Writing – review & editing. XW: Validation, Writing – review & editing. WG: Writing – review & editing, Formal Analysis. DW: Writing – review & editing, Validation. JQ: Writing – review & editing, Formal Analysis. MT: Formal Analysis, Writing – review & editing. YQB: Formal Analysis, Writing – review & editing. HL: Writing – review & editing, Funding acquisition, Methodology, Project administration, Supervision. SW: Funding acquisition, Methodology, Project administration, Writing – review & editing, Conceptualization.
